# The prevalence and correlates of behavioral risk factors for cardiovascular health among Southern Brazil adolescents: a cross-sectional study

**DOI:** 10.1186/1471-2431-12-130

**Published:** 2012-08-25

**Authors:** Valter Cordeiro Barbosa Filho, Wagner de Campos, Rodrigo Bozza, Adair da Silva Lopes

**Affiliations:** 1 Department of Physical Education, Federal University of Santa Catarina, Florianopolis, Brazil; 2 Research Center for Sports and Exercise, Federal University of Parana, Curitiba, Brazil; 3 Department of Physical Education, Federal University of Parana, Curitiba, Brazil

**Keywords:** Adolescent behavior, Epidemiology, Motor activity, Sedentary lifestyle, Eating behavior, Smoking, Alcohol drinking, Brazil

## Abstract

**Background:**

The adoption of health-related behaviors is an important part of adolescence. This study examined the prevalence and correlates of the isolated and simultaneous presence of behavioral risk factors for cardiovascular health (BRFCH) among adolescents in Curitiba, Southern Brazil.

**Methods:**

A cross-sectional study was performed with 1,628 adolescents (aged 11-17.9 years, 52.5% males) that were randomly selected from 44 public schools. Self-report instruments were used to assess the variables. Six BRFCH were analyzed: insufficiently active, excessive TV watching, current alcohol and tobacco use, daily soft drinks consumption and inadequate fruit and vegetable consumption. Sociodemographic and behavioral variables were studied as possible correlates of the presence of BRFCH.

**Results:**

The BRFCH with the highest prevalence were insufficiently active (50.5%, 95% confidence interval [95% CI]: 48.0-52.9) and daily soft drinks consumption (47.6%, 95% CI: 45.1-50.0). Approximately 30% of the adolescents presented three or more BRFCH simultaneously. Girls, adolescents who did not participate in organized physical activity, and who used computer/video games daily were the main high-risk subgroups for insufficiently active. Boys and those who used computer/video games daily were the high-risk subgroups for daily soft drinks consumption. For excessive TV watching, we identified to be at risk those who were from a high economic class, unemployed, and who used computer/video games daily. For current alcohol use, we identified older adolescents, who were from a high economic class and who worked to be at risk. Older adolescents, who worked and who spent little active time during a physical education class were the high-risk subgroups for current tobacco use. For inadequate fruit and vegetable consumption, we identified those who did not participate in organized physical activity to be at risk. Older adolescents, who were from a high economic class, who did not participate in organized physical activity and who used computer/video games daily were the high-risk subgroups for simultaneous BRFCH.

**Conclusions:**

We found a high prevalence of BRFCH among adolescents, both isolated and simultaneously. The correlates of the presence of BRFCH can contribute to healthy policies among Brazilian adolescents, mainly focusing on high-risk subgroups for a health risk behavior.

## Background

Adolescence is an essential period for the development and adoption of health-related behaviors for several reasons [1,2]. First, this period of an individual’s life is characterized by a high susceptibility to environmental factors, such as the media and friends. These factors can have negative effects on the choices for health-related behaviors [3]. Second, many behaviors are established during adolescence and continue into adulthood [2,4]. Finally, some of the inappropriate behaviors that are adopted in adolescence (e.g., alcohol and tobacco abuse) can impact the adolescent’s health status [5] and can contribute to health problems in adulthood [6].

Several behaviors have been linked to cardiovascular diseases, and therefore are considered as behavioral risk factors for cardiovascular health (BRFCH) [7]. For example, insufficiently active is related to the emergence of various cardiovascular problems, including ischemic heart disease and stroke [7,8]. Sedentary behaviors (i.e., too much sitting, as distinct from too little exercise) are also related to mortality from cardiovascular disease [9]. Tobacco use is related to the development of lung cancer and stroke [7]. The use of alcohol can reduce the risk of some cardiovascular diseases, but alcohol abuse, which often occurs in adolescents, has a negative impact on cardiovascular health [7]. Finally, there is also a relationship between eating habits (e.g., low consumption of fruits and vegetables) and the emergence of obesity, high blood pressure, ischemic heart disease and stroke [7,10].

Cardiovascular diseases are prevalent globally, and several behaviors in adolescence may contribute to the emergence of these health problems. Thus, the study of BRFCH in adolescents is very relevant to public health and is encouraged by international institutions [1,11]. In addition, recent evidences suggest that a simultaneous presence of two or more BRFCH can be more harmful to the individual’s health than isolated presence of BRFCH [12]. Therefore, an analysis of the simultaneous presence of BRFCH is needed. Finally, identifying possible correlates of the BRFCH in youth, whether the BRFCH are isolated or simultaneous, can be beneficial for targeting interventions to group who are at risk for engaging in health-related risk behaviors.

The present study aimed to identify the prevalence of BRFCH (insufficiently active, excessive TV watching, current alcohol and tobacco use, daily soft drinks consumption and inadequate fruit and vegetable consumption) among adolescents from public schools in Curitiba, Southern Brazil. Additionally, this study also aimed to analyze the sociodemographic and behavioral correlates of the isolated and simultaneous presence of BRFCH among adolescents.

## Methods

### Sampling procedures

We performed a representative cross-sectional survey, including adolescents who are enrolled in classes from the 6^th^ grade of elementary school to the 2^nd^ year of secondary school in public schools in the metropolitan area of Curitiba, Southern Brazil (Human Development Index of this municipality = 0.856). The sample size for this study was calculated using the following parameters: (i) population of 115,524 adolescents, (ii) confidence level of 95%, (iii) sampling error of 3 percentage points, (iv) prevalence of BRFCH estimated at 50%, (v) design effect of 1.4 [13], and (vi) margin of 30% for possible losses and refusals. Therefore, the study sample was estimated to include 1,800 adolescents.

The sample was selected using a two-stage conglomerate sampling process. In the first stage, five schools (primary sampling unit) were randomly selected in each of the nine microregions of the municipality of Curitiba. Thus, the sampling was stratified by microregion and each school represented one grade for its respective microregion. In case of refusal (three schools), a new school was randomly selected and invited to participate in the study. In the second stage, one to three group-classes (secondary sampling unit) were randomly selected according to the amount of adolescents who was in the respective municipality region.

The selection process generated a sample of 1,812 students. All of these adolescents were evaluated (100% of invited adolescents) after obtaining written informed consent from their parents/guardians. During data analysis, we found that 31 adolescents (1.7%) were outside the age range of interest (18 years and older), 35 adolescents (1.9%) did not complete all of the questionnaire items, and 118 adolescents (6.5%) completed the food frequency questionnaire incorrectly. These adolescents were excluded from the sample. Therefore, the final sample consisted of 1,628 adolescents who were enrolled in 44 public schools in Curitiba, Brazil.

The calculation of the sample statistical power was performed *a posteriori*, considering a confidence level of 95% (α = 0.05) and a power of 80% (β = 0.20). The sample of 1,628 adolescents can identify prevalence ratios above 1.3 as risk and below 0.7 as protection for the BRFCH with a prevalence rate above 20% in the unexposed group. For the prevalence of current tobacco use (approximately 6% in the unexposed group), the sample size of 1,628 adolescents can identify prevalence ratios above 1.6 as risk and below 0.4 for protection.

### Measures and data collection procedures

#### Behavioral Risk Factors for Cardiovascular Health (BRFCH)

Data were collected between February and June 2011. Adolescents completed questionnaires in the classroom to identify the BRFCH and correlate variables to be analyzed in the study. The completion of the questionnaires was supervised by the principal investigator (VCBF), with support from previously trained Physical Education professionals and academics.

Insufficiently active was assessed using a self-report questionnaire that was developed by Bouchard et al. [14], which asked questions pertaining to three days in a usual week (two weekdays and one weekend day). The questionnaire determined an estimation of the time involved in moderate to vigorous physical activities (activities with scores from 6 to 9, as proposed in the original instrument) during a typical week [14]. Based on the current physical activity guidelines, teenagers were considered insufficiently active when they reported less than 420 minutes of physical activity per week [15].

The amount of time spent watching TV and current tobacco use were evaluated by a self-report questionnaire that was used in the Youth Risk Behavior Survey [1]. The questionnaire was developed by the Center for Disease Control and Prevention (CDC) [1] and was previously adapted and validated for the young Brazilian population [16]. We felt that the amount of time watching TV on weekdays and weekend days were two separate issues. The adolescents were considered to be at risk for sedentary behavior (excessive TV watching) when they reported three or more hours of TV watching every day. Cigarette smoking during the month preceding the survey was assessed though one question, and the adolescents were considered to be current tobacco users if they reported consuming at least one cigarette in the last month, regardless of the quantity [1].

Eating behaviors outcomes (intake of soft drinks and fruit and vegetables) and the current alcohol use were estimated through a self-report food frequency questionnaire. This questionnaire was developed for the Brazilian population by Sichieri and Everhart [17] and was adapted for adolescents by Fonseca, Sichieri and Veiga [18]. The adolescents were presented with a list of 80 foods items, and were asked to reported the amount and frequency that they had consumed each item in the previous month [17]. We excluded cases of adolescents who had a total energy consumption over 7000 kcal/day or less than 500 kcal/day because these are rare events among adolescents [19]. The consumption of fruits and vegetables was considered to be inadequate if the adolescents reported consuming fewer than five portions per day of fruits and vegetables. We used this guidelines because of the association between consuming this quantity of fruits and vegetables and an individual’s health status [10], and the guideline is also suggested by the CDC [1]. Soft drinks consumption was considered high if the adolescents reported drinking soft drinks daily, as utilized in the Health Behaviour in School-Aged Children study [20]. Finally, the current alcohol use was considered when the adolescent reported consuming at least one dose of alcoholic beverages during the month preceding the survey, regardless of the quantity [1].

To identify the simultaneous presence of BRFCH and possible correlates, the number of BRFCH in each adolescent was recorded (range of 0-6 BRFCH). In the statistical analysis, three different models were used on the number of simultaneous BRFCH. In the first model, each adolescent was classified as having no BRFCH, or as having one or more BRFCH. In the second model, each adolescent was classified as having less than two BRFCH, or as having two or more BRFCH. In the third model, each adolescent was classified as having less than three BRFCH, or as having three or more BRFCH.

#### Possible correlates of isolated and simultaneous BRFCH

Five sociodemographic variables were analyzed. Gender (boys or girls) and age (11-12.9 years, 13-14.9 years or 15-17.9 years) were analyzed as demographic variables. Occupation status (employed or not employed) was also evaluated. In this study, employment was defined as any activity that contributed to the production of goods or services, including unpaid activities, but excluding household chores in the adolescent’s own residence. Economic class and the head of household’s schooling were also evaluated with the questionnaire of the Brazilian Association of Research Companies [21]. This instrument groups subjects into the economic classes (A1 [richest], A2, B1, B2, C1, C2, D, and E [poorest]), based on a score combining ownership of assets, schooling of head of household, and number of employees in the household. For the purposes of analysis, due to the low sample size in categories D and E (17 adolescents), the economic classes were grouped into three categories: A1 + A2 (best condition), B1 + B2, and C + D + E (worst condition). The head of the household’s schooling (parent or guardian who is the chief financial officer and has the highest educational level of the household) was classified into four categories: less than 4 years, 4-8 years, 9-11 years, and greater than 12 years.

Additionally, four behavioral variables were also analyzed. Each adolescent reported the number of physical education (PE) classes in a habitual week, and the answers were classified in three categories (no class/dispensed, 1-2 or more classes, or three classes). The adolescent also answered an item about the time spent in physical activities during a PE class (in Brazil, each PE class has a mean time of approximately 50 minutes). The answers were classified into three categories: (i) did not participate, (i) less than 30 minutes, and (iii) more than 30 minutes. Finally, the adolescents responded to two dichotomous questions about the participation in organized physical activity (yes or no) and the daily use of computer/video games during leisure time (yes or no).

### Statistical analysis

The absolute and relative frequency were used for the description of variables. A 95% confidence interval (95% CI) was calculated for the prevalence of each BRFCH among adolescents. The prevalence of isolated and simultaneous BRFCH was also calculated for the independent variables. In the unadjusted analysis, the proportion differences between categories were evaluated by a Chi-square test for linear trend or heterogeneity. In the multivariate analyses, the Poisson regression with robust variance [22] was used to calculate the adjusted prevalence ratios. Before performing the adjusted analyses, we performed tests for collinearity among the potential correlates, but none of them suggested high levels of multicollinearity. Thus, all independent variables were used in the multivariate model. Statistical analyzes were performed using Stata 10.1 (Stata Corp., College Station, USA) and took into account the selection strategy of cluster sampling using the command "svy". The level of significance was 5% for two-tailed tests.

### Ethical considerations

The Research Ethics Committee of the Federal University of Parana approved this study (CAAE: 5371.0.000.091-10). Each school has given formal permission to the data collection at school. Each adolescent’s parent/guardian provided written informed consent for participation in this study.

## Results

The mean age of the sample was 14.3 years, with a standard deviation of 1.6 years. The sample had a higher participation of boys (52.5%), adolescents aged from 13-14.9 years (38.0%), adolescents in the B1 + B2 economic class (62.1%), adolescents who were unemployed (82 3%) and adolescents whose head of the family had 9-11 years of education (39.9%). Additionally, most adolescents had three PE classes per week (54.7%) and actively participated for 30 minutes or more in a PE class (43.9%). However, many adolescents did not participate in organized physical activity (65.7%) and used computer/video games daily (67.4%).

**Figure 1:**
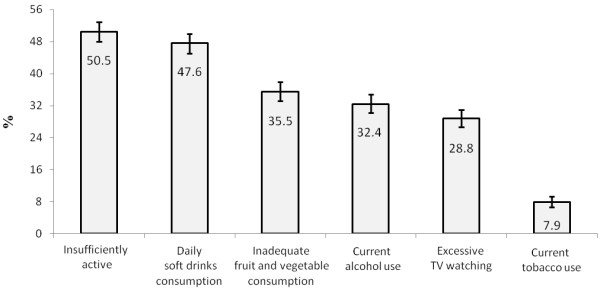
Prevalence and 95% confidence interval of behavioral risk factors for cardiovascular health among Brazilian adolescents.

The BRFCH with the highest prevalence among the adolescents was insufficiently active (50.5%, 95% CI: 48.0-52.9), followed by daily soft drinks consumption (47.6%, 95% CI: 45.1-50.0). Inadequate fruit and vegetable consumption and current alcohol use were present in 35.5% (95% CI: 33.2-37.9) and 32.4% (95% CI: 30.2-34.8) of the adolescents, respectively. Three out of 10 adolescents spent an excessive time watching TV (28.8%, 95% CI: 26.6-31.0), whereas 7.9% (95% CI: 6.7-9.3) of adolescents consumed tobacco in the previous month (Figure 1).

The prevalence of BRFCH based on the independent variables is presented in Table 1. In the bivariate analysis, boys were associated with higher prevalence of excessive TV watching and daily soft drinks consumption. Girls were associated with a higher prevalence of insufficiently active and inadequate fruit and vegetable consumption. Age was associated with current alcohol and tobacco use, and an increasing trend in the prevalence of alcohol and tobacco use with age was seen.

**Table 1:** Prevalence of behavioral risk factors for cardiovascular health according to the independent variables among Brazilian adolescents

		**Behavioral risk factors for cardiovascular health (%)**					
**Variables**	**n (%)**	**Insufficiently**	**Excessive**	**Current**	**Current**	**Daily**	**Inadequate fruit**
		**active**	**TV watching**	**alcohol**	**tobacco**	**soft drinks**	**and vegetable**
				**use**	**use**	**consumption**	**consumption**
Gender							
Boys	773 (52.5)	34.3*	31.4*	32.0	6.9	53.3*	33.1*
Girls	855 (47.5)	65.1	26.3	32.9	8.9	42.5	37.7
Age groups							
11-12.9 years	446 (27.4)	46.0	26.2	15.9**	2.0**	44.4	32.3
13-14.9 years	618 (38.0)	52.4	31.1	35.1	9.1	50.0	37.2
15-17.9 years	564 (34.6)	52.0	28.2	42.6	11.3	47.5	36.2
Economic class							
A1 + A2 (best condition)	93 (5.7)	37.6	40.9**	49.5**	11.8	58.1**	36.6
B1 + B2	1011 (62.1)	53.0	29.6	31.0	7.8	50.0	35.2
C + D + E (worst condition)	524 (32.2)	47.9	25.0	32.3	7.4	41.2	35.9
Head of household’s schooling							
≤ 4 years	158 (9.7)	48.1	19.0**	36.1	7.6	46.2	36.7
4-8 years	438 (26.9)	50.2	26.0	33.1	7.3	48.6	34.5
9-11 years	649 (39.9)	49.3	31.7	32.8	8.6	49.6	35.6
≥ 12 years	383 (23.5)	53.8	30.8	29.5	7.6	47.6	36.0
Employment status							
No	1340 (82.3)	51.3	30.2*	29.4*	6.3*	46.4*	36.0
Yes	288 (17.7)	46.9	21.9	46.5	15.3	53.1	33.3
Weekly PE class							
3 classes	890 (54.7)	47.1**	27.5	30.4**	7.8	48.8**	36.1
1-2 classes	554 (34.0)	53.1	29.1	32.7	7.8	48.0	32.7
No class/dispensed	184 (11.3)	59.2	33.7	41.3	9.2	40.8	41.3
Active time during a PE class							
30 or more minutes	715 (43.9)	41.8**	28.7	28.2**	5.5**	49.7**	33.1
Until 30 minutes	647 (39.7)	56.4	27.5	33.8	10.2	47.9	36.6
Did not participate/dispensed	266 (16.3)	59.4	32.0	40.2	9.0	41.4	39.1
Organized physical activity							
Yes	559 (34.3)	27.0**	29.7	30.4	8.2	51.7*	27.7*
No	1069 (65.7)	62.8	28.3	33.5	7.8	45.5	39.6
Use of computer/video games daily during leisure time							
No	531 (32.6)	49.7	2.6*	27.5*	6.6	39.9*	34.3
Yes	1097 (67.4)	50.9	41.4	34.8	8.6	51.3	36.1

PE = physical education.

* Qui-square test for heterogeneity, p < 0.05.

** Qui-square test for linear trend, p < 0.05.

Economic class was positively associated with daily soft drinks consumption and current alcohol and tobacco use. There was a positive association between excessive TV watching and the head of household’s schooling. Employed adolescents had a higher prevalence of current alcohol and tobacco use and daily soft drinks consumption as well as a lower prevalence of excessive TV watching (Table 1).

There was a negative association between the frequency of PE class and insufficiently active and current alcohol use. There was also a negative association between time spent active in PE class and insufficiently active and current alcohol and tobacco use. Adolescents who did not participate in organized physical activity had a higher prevalence of insufficiently active and inadequate fruit and vegetable consumption. Finally, daily computer/video games use was associated with a higher prevalence of excessive TV watching, current alcohol use and daily soft drinks consumption (Table 1).

In the adjusted analysis (Table 2), we found that girls were a high-risk subgroup for the insufficiently active. However, the girls were a protective subgroup for daily soft drinks consumption. Age remained positively associated with the current alcohol and tobacco use among adolescents. Economic class was positively associated with excessive TV watching and current alcohol use. Adolescents who worked were a high-risk subgroup for current alcohol and tobacco use, but were a protective subgroup for the excessive TV watching.

**Table 2:** Adjusted prevalence ratios (PR) of behavioral risk factors for cardiovascular health and correlates among Brazilian adolescents

		**Behavioral risk factors for cardiovascular health**					
**Variables**	**n**	**Insufficiently active**	**Excessive TV watching**	**Current alcohol use**	**Current tobacco use**	**Daily soft drinks consumption**	**Inadequate fruit and vegetable consumption**
Gender							
Boys	773	Reference	Reference	Reference	Reference	Reference	Reference
Girls	855	1.60 (1.42- 1.81) **	0.96 (0.83-1.11)	1.06 (0.91-1.24)	1.41 (0.92-2.15)	0.84 (0.74-0.96) **	1.06 (0.92-1.21)
Age groups							
11-12.9 years	446	Reference	Reference	Reference	Reference	Reference	Reference
13-14.9 years	618	1.11 (0.98-1.26)	1.00 (0.83-1.21)	2.08 (1.57-2.77) **	4.25 (2.40-7.54) **	1.12 (0.96-1.30)	1.15 (0.96-1.37)
15-17.9 years	564	1.08 (0.95-1.23)	0.91 (0.74-1.13)	2.37 (1.74-3.23) **	4.96 (2.44-10.06) **	1.07 (0.92-1.25)	1.10 (0.89-1.37)
Economic class							
A1 + A2 (best condition)	93	Reference	Reference	Reference	Reference	Reference	Reference
B1 + B2	1011	1.30 (0.98-1.72)	0.74 (0.59-0.93) *	0.56 (0.43-0.72) **	0.64 (0.37-1.11)	0.89 (0.76-1.03)	0.90 (0.69-1.19)
C + D + E (worst condition)	524	1.15 (0.88-1.51)	0.73 (0.56-0.95) *	0.54 (0.39-0.75) **	0.62 (0.31-1.21)	0.73 (0.59-0.89) **	0.90 (0.68-1.20)
Head of household’s schooling							
≤ 4 years	158	Reference	Reference	Reference	Reference	Reference	Reference
4-8 years	438	1.00 (0.83-1.20)	1.21 (0.88-1.66)	0.91 (0.71-1.15)	0.98 (0.45-2.14)	1.00 (0.82-1.23)	0.92 (0.72-1.18)
9-11 years	649	0.99 (0.82-1.20)	1.32 (0.95-1.83)	0.90 (0.72-1.11)	1.17 (0.58-2.35)	0.90 (0.72-1.11)	0.96 (0.75-1.24)
≥ 12 years	383	1.09 (0.89-1.33)	1.16 (0.82-1.64)	0.75 (0.56-1.00)	1.02 (0.49-2.14)	0.92 (0.74-1.14)	0.97 (0.76-1.25)
Employment status							
No	1340	Reference	Reference	Reference	Reference	Reference	Reference
Yes	288	0.95 (0.83-1.09)	0.77 (0.61-0.97) *	1.36 (1.15-1.62) **	2.05 (1.44-2.91) **	1.12 (0.98-1.28)	0.92 (0.76-1.11)
Weekly PE class							
3 classes	890	Reference	Reference	Reference	Reference	Reference	Reference
1-2 classes	554	1.07 (0.97-1.20)	0.98 (0.82-1.17)	0.97 (0.81-1.16)	0.85 (0.53-1.35)	0.96 (0.87-1.06)	0.89 (0.74-1.07)
No class/dispensed	184	1.17 (0.93-2.00)	0.93 (0.63-1.36)	1.02 (0.76-1.37)	0.96 (0.38-2.45)	0.85 (0.64-1.14)	1.18 (0.81-1.72)
Active time during a PE class							
30 or more minutes	715	Reference	Reference	Reference	Reference	Reference	Reference
Until 30 minutes	647	1.13 (0.98-1.29)	1.01 (0.88-1.17)	1.17 (0.98-1.39)	1.76 (1.14-2.72) *	1.02 (0.90-1.14)	1.04 (0.87-1.25)
Did not participate/dispensed	266	1.03 (0.98-1.29)	1.21 (0.87-1.69)	1.12 (0.81-1.55)	1.21 (0.49-3.00)	0.95 (0.76-1.20)	0.92 (0.65-1.31)
Organized physical activity							
Yes	559	Reference	Reference	Reference	Reference	Reference	Reference
No	1069	1.99 (1.74-2.27) **	1.04 (0.89-1.21)	1.05 (0.90-1.22)	0.79 (0.57-1.10)	0.95 (0.86-1.05)	1.40 (1.18-1.67) **
Use of computer/video games daily during leisure							
No	531	Reference	Reference	Reference	Reference	Reference	Reference
Yes	1097	1.10 (1.02-1.18) *	15.25 (8.94-26.01)**	1.20 (0.99-1.46)	1.22 (0.81-1.86)	1.24 (1.09-1.40) **	1.06 (0.89-1.26)

PR = Prevalence ratio adjusted for all other variables contained in the regression model. PE = physical education. 95% CI = 95% confidence interval.

* P < 0.05 (Wald test).

** P < 0.01 (Wald test).

Adolescents who spent little active time in PE class (less than 30 minutes) were a high-risk subgroup for current tobacco use. The lack of participation in organized physical activity was associated with a higher prevalence of insufficiently active and inadequate fruit and vegetable consumption. Finally, adolescents who used computer/video games daily were a high-risk subgroup for insufficiently active, excessive TV watching and daily soft drinks consumption. In the multivariate analysis, head of household’s schooling and frequency of PE class were not associated with BRFCH (Table 2).

**Figure 2:**
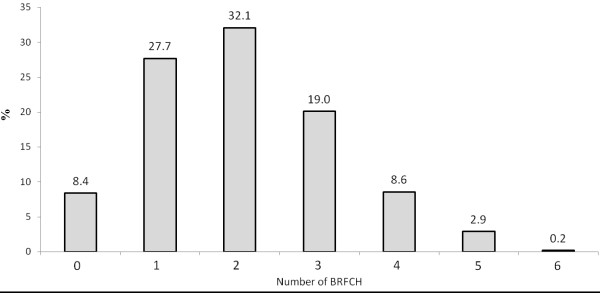
Prevalence of the simultaneous behavioral risk factors for cardiovascular health (BRFCH) among Brazilian adolescents.

Only 8.4% of the adolescents did not show any BRFCH (Figure 2). In contrast, approximately 60% of the adolescents presented with one or two BRFCH simultaneously. Three out of 10 adolescents presented with three or more BRFCH simultaneously. Less than 1% of the adolescents presented with six BRFCH (Figure 2).

In the adjusted analysis for the simultaneous BRFCH (Table 3), girls were a high-risk subgroup for the simultaneous presence of three or more BRFCH. Older age was associated with the presence of BRFCH (model 1) and with the simultaneous presence of several BRFCH (model 2 and 3). For example, the prevalence of three or more BRFCH was approximately 70% higher in adolescents aged 13-14.9 years and 15-17.9 years compared to their younger peers (11-12 years).

**Table 3:** Adjusted prevalence ratios (PR) for the simultaneous behavioral risk factors for cardiovascular health and correlates among Brazilian adolescents

**Variables**		**Model 1**		**Model 2**		**Model 3**	
		**≥ 1 BRFCH** ***		**≥ 2 BRFCH** ***		**≥ 3 BRFCH** ***	
		%	RP (95% CI)	%	RP (95% CI)	%	RP (95% CI)
Gender							
Boys	773	90.2	Reference	61.6	Reference	27.7	Reference
Girls	855	93.0	1.03 (0.98-1.08)	66.1	1.06 (0.98-1.14)	35.7	1.25 (1.06-1.46)**
Age years``							
11-12.9 years	446	87.7	Reference	52.5	Reference	19.7	Reference
13-14.9 years	618	92.4	1.04 (1.00-1.08)*	66.0	1.21 (1.10-1.34)**	37.1	1.69 (1.27-2.25)**
15-17.9 years	564	94.0	1.06 (1.01-1.10)*	70.7	1.28 (1.14-1.42)**	35.8	1.57 (1.14-2.14)**
Economic class							
A1 + A2 (best condition)	93	92.5	Reference	75.3	Reference	43.0	Reference
B1 + B2	1011	92.4	0.98 (0.92-1.04)	66.2	0.85 (0.75-0.96)*	31.9	0.69 (0.54-0.90)**
C + D + E (worst condition)	524	90.1	0.95 (0.88-1.02)	57.6	0.74 (0.63-0.86)**	29.8	0.65 (0.48-0.89)**
Head of household’s schooling							
≤ 4 years	158	92.4	Reference	60.8	Reference	29.7	Reference
4-8 years	438	92.0	0.98 (0.94-1.03)	63.2	0.99 (0.86-1.15)	30.6	0.96 (0.72-1.28)
9-11 years	649	91.7	0.97 (0.92-1.03)	64.1	0.97 (0.84-1.11)	32.8	0.98 (0.71-1.36)
≥ 12 years	383	90.9	0.95 (0.90-1.02)	65.8	0.94 (0.82-1.07)	32.6	0.93 (0.67-1.30)
Employment status							
No	1340	91.2	Reference	63.5	Reference	30.7	Reference
Yes	288	93.8	1.02 (0.98-1.06)	66.0	1.01 (0.92-1.09)	37.2	1.23 (1.02-1.49)*
Weekly PE class							
3 classes	890	90.4	Reference	61.1	Reference	30.8	Reference
1-2 classes	554	92.2	1.01 (0.98-1.04)	65.9	1.02 (0.94-1.10)	30.7	0.90 (0.75-1.08)
No class/dispensed	184	95.7	1.06 (0.99-1.14)	67.7	0.98 (0.81-1.20)	40.8	1.14 (0.79-1.63)
Active time during a PE class							
30 or more minutes	715	89.0	Reference	59.4	Reference	26.7	Reference
Until 30 minutes	647	92.6	1.01 (0.97-1.06)	65.8	1.06 (0.97-1.16)	34.9	1.18 (0.95-1.46)
Do not participate	266	94.0	0.97 (0.90-1.04)	71.4	1.08 (0.90-1.29)	38.3	1.00 (0.71-1.41)
Organized physical activity							
Yes	559	87.7	Reference	55.6	Reference	21.8	Reference
No	1069	93.7	1.08 (1.02-1.11)**	68.3	1.21 (1.10-1.33)**	37.1	1.62 (1.32-1.97)**
Use of computer/video games daily during leisure time							
No	531	87.2	Reference	50.3	Reference	17.7	Reference
Yes	1097	93.8	1.08 (1.03-1.12)**	70.6	1.38 (1.26-1.51)**	38.7	2.17 (1.74-2.71)**

PR = Prevalence ratio adjusted for all other variables contained in the regression model. PE = physical education. 95% CI = 95% confidence interval.

* P < 0.05 (Wald test).

** P < 0.01 (Wald test).

*** Reference condition (outcome) considered in this regression model.

Economic class was positively associated with the simultaneous presence of BRFCH (models 2 and 3). Employed adolescents were also associated with the presence of three or more BRFCH. Finally, adolescents who did not participate in organized physical activity and who used computer/video games daily were high-risk subgroups for simultaneous BRFCH. For example, the prevalence of three or more BRFCH was 60% higher among adolescents who did not participate in organized physical activity compared to their peers who participated in organized physical activity. Likewise, the prevalence of three or more BRFCH in adolescents who used computer/video games daily was twice greater compared with those who did not use these electronic resources daily (Table 3).

## Discussion

The results of this study showed the main BRFCH that are adopted by adolescents in public schools in Curitiba, Southern Brazil. Insufficiently active and daily soft drinks consumption and were the most common BRFCH among adolescents, with prevalence rates close to 50% (Figure 1). Additionally, approximately 30% of the adolescents had three or more BRFCH simultaneously, which indicates a high-risk situation for the adolescent health (Figure 2). Given this evidence, we suggest the urgent need for interventions to reduce BRFCH in Brazilian adolescents.

The prevalence of daily soft drinks consumption was higher than those observed in other studies with Brazilian adolescents [23,24]. Moreover, the prevalence of daily soft drinks consumption obtained in the present study was higher than the estimate for American [1] and European [20,25] adolescents. This study also found a prevalence of insufficiently active higher than another study using the current physical activity guidelines for adolescents [26]. These differences may be related to the use of different instruments to estimate BRFCH, mainly for soft drinks consumption. However, sociocultural and behavioral distinctions (e.g., regional distinctions in encouraging the consumption of healthy food and physical activity) may explain the differences in prevalence between these BRFCH.

There were similarities in the prevalence of current alcohol and tobacco use between adolescents from Curitiba and from other regions of Brazil [27,28]. However, the prevalence rates for these BRFCH were higher than those observed in a survey of adolescents from all 27 Brazilian capitals (27.3% and 6.3% for current alcohol and tobacco use, respectively) [29]. These results indicate that adolescents from public schools in Curitiba had a consumption pattern that is different from the national pattern.

The prevalence of excessive TV watching in adolescents from Curitiba was lower than that found in other Brazilian studies [29,30], which examined TV watching during the weekdays. Importantly, this study considered the excessive TV watching when the adolescent reported watching TV for three or more hours every day in a typical week. If this study had used a cutoff point of three or more hours only on weekdays, the estimated prevalence (58.2%, data not shown in tables) would still be lower than national estimates for Brazilian adolescents [29]. However, these estimates were higher than those found for American [1] and European [25] adolescents. Therefore, evidence suggests that the sedentary habit represents a BRFCH that is increasingly present in the young population, and Brazilian adolescents have alarming estimated rates of this BRFCH. Interventions that seek to promote healthy lifestyles in this population should focus on reducing sedentary behaviors among young people.

It is important to highlight that the inadequate fruit and vegetable consumption (≤ 5 portions per day) had estimates that were below those found in previous studies [1,31]. These studies used food frequency questionnaires to estimate the intake of fruits and vegetables. Therefore, it is clear that the habit of eating small amounts of fruits and vegetables has lower estimates in adolescents in Curitiba, Brazil. The environmental and demographic factors (e.g., availability of fruits and vegetables throughout the year, encouraging parental consumption and price of food) that contribute to the adoption of this healthy habit must be analyzed in future research. The study of these variables can contribute to the development of actions to promote healthy eating among young people from Brazil and other countries.

A concerning result was the high prevalence of adolescents with a simultaneous presence of BRFCH. In particular, 30.7% of the adolescents presented with three or more BRFCH simultaneously (Figure 2). The comparison of the estimates of the present study with data from other localities is limited, because these studies used different outcomes for the simultaneous analysis of BRFCH. However, because simultaneous presence of risk behaviors may be more harmful to an individual’s health [12], combating both isolated and simultaneous BRFCH is an important focus of intervention for promoting health among Brazilian youth.

Girls and older adolescents were identified as two high-risk subgroups for several BRFCH, mainly regarding the simultaneous presence of BRFCH (Tables 2 and 3). There is consistency between studies indicating that older adolescents tend to adopt several BRFCH, such as alcohol [32-34] and tobacco [35] use, or the simultaneous presence of several BRFCH [36]. However, there are differences among studies regarding the association between gender and BRFCH. Previous studies have highlighted that boys represent a high-risk subgroup for adopting inappropriate health behaviors, such as daily soft drinks consumption [20], current alcohol use [37,38] and excessive TV watching [20]. Furthermore, other studies have suggested that girls represent a high-risk subgroup for the BRFCH of tobacco use [27,37] and insufficiently active [1,20,36,38]. These gender differences in BRFCH may be related to historical and social conditions surrounding the concepts of identity that are inherent in males and females [37]. The concepts of identity and environmental factors contribute to the preference and choice for some gender-specific BRFCH (e.g., more physical activity practice among boys, while girls prefer low energy expenditure activities) [20,27,36]. Thus, gender is an important variable in an intervention design for prevention of adolescent BRFCH. The gender subgroup at risk is highly dependent on the BRFCH target for the intervention program.

Economic class was positively associated with some BRFCH (excessive TV watching, current alcohol use, and daily soft drinks consumption), as well as with the simultaneous presence of one or more BRFCH in adolescents (Tables 2 and 3). Previous studies also showed an association between economic status and BRFCH among adolescents [36,38-40]. However, the direction of this association varied according to the BRFCH. For example, a study performed in 28 European and North American countries found a positive association between economic status and alcohol use and excessive TV watching, but there was an inverse association for daily vegetable consumption [40]. Another survey including Korean adolescents also showed a positive association between economic status and alcohol use, but an inverse association was obtained for insufficiently active [38]. Furthermore, previous studies highlighted that the direction of the association may vary from region to region [39,40]. Given the evidence of the present study, adolescents whose families have better economic conditions seem to represent a subgroup that also should be carefully observed within the school environment in an effort to reduce these poor habits among adolescents.

Employment was significantly and positively associated with current alcohol and tobacco use, and the presence of three or more BRFCH among adolescents in this study. This association was also confirmed elsewhere [41,42]. There are several features that might explain the relationship between these variables. The responsibilities that adolescents acquire by being part of labor market (i.e., most of them earn their own money and then have less social limits imposed by parents/guardians), contributes to their adopting inappropriate health habits [41]. This employment situation is also characterized by the social involvement of adolescents with adults, which can encourage the young to imitate adult behavior. However, the mechanism that explains the association between employment status and BRFCH in adolescents may vary depending on the outcome studied [41]. New studies are required to explain this association. Importantly, there remains the need for parents and health professionals to pay special attention to individuals who need to work during adolescence.

The present study also indicated a relationship between behavioral factors and some BRFCH. For example, adolescents who did not participate in organized physical activity were a high-risk subgroup for adopting BRFCH (insufficiently active and inadequate fruit and vegetable consumption). This subgroup of adolescents was also more prone to the simultaneous presence of BRFCH. Additionally, adolescents who spent little active time in a PE class (less than 30 minutes) represented a high-risk subgroup for current tobacco use. Evidence of an association between components of physical activity and BRFCH has been previously highlighted [30,43-45]. The encouragement of physical activity inside and outside of the school can play an important role not only in protecting against cardiovascular health problems, but also in the healthy lifestyle based on different behaviors.

Finally, the present study emphasized the association between the use of electronic resources (computer and video games) during leisure time and the presence of BRFCH (insufficiently active, excessive TV watching and daily soft drinks consumption) among adolescents. The association between the use of computer/video games and insufficiently active could be related to the frequent preference of adolescents for sedentary activities during leisure times rather than physical activities [43,45]. The association between sedentary behavior and inappropriate eating habits (e.g., frequent consumption of fried foods and sugary drinks) was also highlighted in a systematic review [46] and is related to the consumption of these foods during sedentary activities.

Additionally, we found that the daily use of computer/video games is also significantly associated with the simultaneous presence of BRFCH. The association of the excessive use of media resources during leisure time and the simultaneous presence of inappropriate health behaviors in adolescence was also previously highlighted [47]. Based on this evidence, it is clear that sedentary behavior may be a factor that encourages the adoption of other BRFCH. With increasing of technological items (e.g., computer, cell phone, and TV) in the daily lives of adolescents, sedentary leisure practices can contribute to high prevalence rates of other BRFCH among adolescents. Therefore, interventions to promote health in young Brazilians should focus on reducing sedentary behaviors. These intervention actions can contribute to the reduction of other behaviors that are associated with the development of cardiovascular problems, such as insufficiently active and high consumption of energy-rich foods.

A strength of this study was the analysis of six important BRFCH in a representative sample of adolescents from public schools in a major Brazilian municipality. The public schools in Curitiba serve approximately 80% of all students across the city and are the main focus of health promotion public policies in Brazil. Additionally, the identification of correlates of the isolated and simultaneous presence of BRFCH among adolescents contributed to the identification of high-risk subgroups for adopting these behaviors. This evidence can guide interventions that promote a healthy lifestyle in adolescents.

The present study also had limitations. The first limitation is related to the use of self-report methods for the identification of variables, especially the BRFCH. Although the self-report questionnaires are often used in epidemiological studies with adolescents [1,20,25,29], these instruments tend to have less accuracy in identifying BRFCH, such as the underestimation of alcohol and tobacco use, as well as the overestimation of physical activity (which underestimates the prevalence of insufficiently active). Therefore, the presence of some BRFCH may be even greater than we identified in this study. The second limitation is related to the fact that the sample was extracted only from adolescents from public schools, thus limiting the extrapolation of the results to other groups of adolescents. The final limitation is related to the use of a cross-sectional design to indicate associations between the variables, as an inherent characteristic of the cross-sectional design is the possibility of reverse causality. These limitations demonstrate the need for caution when interpreting the results of this study.

## Conclusions

The results of this study indicate that the BHFCH with the highest prevalence in adolescents in Curitiba were insufficiently active and daily soft drinks consumption. A high prevalence of the simultaneous presence of BRFCH among adolescents was found, which indicates that a large proportion of adolescents had at least one behavior that is harmful to cardiovascular health. The main high-risk subgroups for the isolated and simultaneous presence of BRFCH among adolescents were highlighted in this study. For example, older adolescents, who were from high economic class, who did not participate in organized physical activity and who used computer/video games daily were the high-risk subgroups for simultaneous BRFCH. Evidence from this study calls attention to the urgent need for interventions focused on reducing inappropriate lifestyle habits in Brazilian adolescents. Actions to promote a healthy lifestyle should be targeted to subgroups where these behaviors are more prevalent.

## Abbreviations

95% IC: 95% confidence interval; BRFCH: Behavioral risk factors for cardiovascular health; CDC: Center for Disease Control and Prevention; PE: Physical education; PR: Prevalence ratio.

## Competing interests

The authors declare that they have no competing interests.

## Authors’ contributions

All authors were involved in the conception and design of the study. VCBF and RB were involved in the data collection. VBCF conducted the data analysis and drafted the manuscript. All authors read and approved the final manuscript.

## Pre-publication history

The pre-publication history for this paper can be accessed here:

http://www.biomedcentral.com/1471-2431/12/130/prepub
